# Frequency of serological markers of rheumatoid arthritis in patients with IgA anti‐β2 glycoprotein I antibodies

**DOI:** 10.1002/jcla.24537

**Published:** 2022-06-06

**Authors:** Sarra Melayah, Mariem Ghozzi, Amani Mankaï, Fatma Mechi, Ibtissem Ghedira

**Affiliations:** ^1^ Laboratory of Immunology Farhat Hached Hospital Sousse Tunisia; ^2^ Department of Immunology, Faculty of Pharmacy Monastir University Monastir Tunisia; ^3^ LR12SP11, Biochemistry Department Sahloul University Hospital Sousse Tunisia; ^4^ Research Unit "Epidemiology and Immunogenetics of Viral Infections, LR14SP02" Sahloul University Hospital Sousse Tunisia; ^5^ High School of Sciences and Techniques of Health Tunis El Manar University Tunis Tunisia; ^6^ Research Unit "Obesity: Etiopathology and Treatment, UR18ES01" National Institute of Nutrition and Food Technology Tunis Tunisia

**Keywords:** anti‐cyclic citrullinated peptides antibodies, antiphospholipid syndrome, anti‐β2 glycoprotein I antibodies, rheumatoid arthritis, rheumatoid factors

## Abstract

**Aim:**

To determine the frequency of serological markers of RA in patients with anti‐β2 glycoprotein I antibodies (aβ2GPI) of IgA isotype.

**Material and Methods:**

A retrospective study was conducted on 67 patients with aβ2GPI‐IgA. Ninety healthy blood donors (HBD) were used as a control group. IgG anti‐cyclic citrullinated peptides antibodies (CCP‐Ab) and rheumatoid factors (RF) IgG, IgA, and IgM were detected by enzyme‐linked immunosorbent assay (ELISA).

**Results:**

Seventeen patients and eight HBD had CCP‐Ab and/or RF (25.4% vs. 8.9%, *p* = 0.005, CI 95% [14.95; 35.79], odds ratio = 3.5). The frequency of CCP‐Ab was significantly higher in patients than in healthy subjects (14.9% vs. 3.3%, *p* = 0.009). IgA isotype of RF was significantly higher in patients than in controls (7.5% vs. 0%, *p* = 0.02). In male patients, CCP‐Ab and/or RF were more frequent than in healthy male subjects (37.5% vs. 11.8%, *p* = 0.02). In patients, no correlation was found between the levels of aβ2GPI‐IgA and CCP‐Ab (*r* = 0.082, *p* = 0.51). There was no correlation between the level aβ2GPI‐IgA and the level of the isotypes of RF (IgG, IgA, and IgM) in patients (*r* = 0.1, *p* = 0.37; *r* = 0.17, *p* = 0.17 and *r* = 0.07, *p* = 0.59 respectively).

**Conclusion:**

Frequencies of CCP‐Ab and RF are high in patients with aβ2GPI‐IgA suggesting that these patients are susceptible to developing RA.

## Graphical abstract

Frequency of CCP‐Ab and RF in patients with IgA anti‐β2 glycoprotein I antibodies.

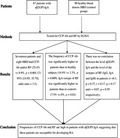

## 1 INTRODUCTION

Beta 2 Glycoprotein I (β2GPI) is a plasma protein with a circulating concentration of 0.2 mg/ml. This molecule plays a key role in hemostasis, homeostasis, and immunity.^1^ Antibodies against β2GPI (aβ2GPI) have been associated with antiphospholipid syndrome (APS) pathogenicity. Antiphospholipid syndrome is formally defined as the association of arterial/venous thrombosis and/or pregnancy losses in patients with persistent antiphospholipid antibodies (aPL).^2^ These autoantibodies include not only aβ2GPI but also anticardiolipin antibodies (aCL) and lupus anticoagulant. Although only IgG and IgM isotypes of aCL and aβ2GPI are included in the 2006 APS classification criteria,^3^ current evidence suggests that aβ2GPI‐IgA may be involved in the pathophysiology of APS.^4^, ^5^, ^6^ In fact, it has been shown that isolated aβ2GPI‐IgA positivity (without IgG or IgM aCL/aβ2GPI and LA) is associated with venous thrombosis and pregnancy morbidity.^4^ However, aβ2GPI‐IgA was demonstrated in autoimmune diseases other than APS.^7^, ^8^, ^9^

Rheumatoid arthritis (RA) is a chronic and systemic autoimmune disease. It is characterized by synovial inflammation that can lead to irreversible joint damage and disability when not managed.^10^ Biomarkers of RA available for routine clinical use are rheumatoid factors (RF) and anti‐cyclic citrullinated peptides (CCP‐Ab). These autoantibodies are found in up to 80% and 95% of RA patients, respectively.^11^

Common links between RA and APS have been reported. Glycosylation is implicated in the synthesis of highly pathogenic forms of antibodies to citrullinated proteins.^12^ Moreover, dysregulation of glycosylation could also generate antibodies of APS.^1^ Microparticles are considered mediators of cellular cross‐talk inflammatory disease and high levels of microparticles have been found in both APS and RA.^13^ Regarding cytokines, tumor necrosis factor (TNF) is of central importance in RA pathogenesis.^14^ On the other hand, aβ2GPI induces monocyte release of TNF.^15^ Another main link is the genetic predisposition, which is important in the development of antibodies of APS^16^ and plays a role in RA risk, severity, and progression of this disease.^14^, ^17^ Common genes for the two diseases include partly HLA system (HLA‐DR4).^14^, ^16^ In addition, an elegant recent study revealed that aβ2GPI‐IgA is associated with the development and progression of coronary atherosclerosis in RA and predicts formation and persistence of high‐risk mixed plaques.^18^

Since many similarities exist between APS and RA and we have previously demonstrated that aβ2GPI‐IgA is frequent in patients with RA.^9^ The objective of the present study was therefore to know if the vice versa is true by determining the serological markers of RA in patients who have aβ2GPI‐IgA.

## 2 PATIENTS AND METHODS

### 2.1 Patients and controls

This retrospective study included sera of 67 patients collected from the database of our laboratory. The inclusion criteria of our study were positivity of aβ2GPI‐IgA. Patients with other autoimmune diseases were excluded, particularly systemic lupus erythematosus and Sjögren syndrome. Antinuclear antibodies (ANA) were negative for all patients. Sera were collected between January 2018 and December 2019 from four hospitals in the center of Tunisia. Patients were consulted for suspicion of APS. The diagnosis of APS could not be confirmed because we do not have the second sample for the antiphospholipid antibodies assay. Medical records of the patients were retrospectively reviewed, and clinical events evoking APS were noted (Table 1).

**TABLE 1:** Clinical manifestations of patients with aβ2GPI

Clinical manifestations	Number of patients
Cirrhosis	3
Deep vein thrombosis	11
Arterial thrombosis	7
Thrombophlebitis	2
Stroke	3
Pregnancy loss	4
Thrombocytopenia	3
Ulcers	1
Pulmonary embolism	1
Budd‐Chiari syndrome	1
Finger gangrene	2
Migraine	1
Hemolytic anemia	1
Cutaneous lesions	4
Purpura	2
Vasculitis	3
Dyspnea	1
Neurologic manifestations	5
Renal manifestation	1
Cardiac manifestations	2

Sera from 90 healthy blood donors (HBD) served as the control group.

All sera were stored at −80°C until the use. The ethical committee of our hospital approved the study.

### 2.2 Methods

#### 2.2.1 Anticardiolipin antibodies assays

The serum samples were evaluated for aCL‐IgG, IgA, and IgM using a commercial enzyme‐linked immunosorbent assay (ELISA; Orgentec Diagnostika®, Mainz, Germany). Results were expressed as arbitrary units with a cutoff for positivity of 10 U/ml for IgA and IgG and 7 U/ml for IgM following the manufacturer's instructions.

#### 2.2.2 Anti‐β2 glycoprotein I antibodies assays

The determination of aβ2GPI IgG, IgA, and IgM was carried out with a commercial ELISA (Orgentec Diagnostika®) using a purified human β2GPI. The results were expressed as arbitrary units with a cutoff for positivity of 8 U/ml following the manufacturer's instructions.

#### 2.2.3 Rheumatoid factors and CCP‐Ab assays

Serum samples were evaluated for IgG, IgA, and IgM rheumatoid factors (RF) by using an ELISA (Orgentec Diagnostika®) according to the manufacturer's instructions. CCP‐Ab was detected by using an available second‐generation ELISA (Euroimmun®, Lubeck, Germany). Optimal cut‐off values were determined by plotting sensitivity against 1‐specificity to receiver operating characteristic (ROC) curve as we have described in our previous studies.^19^, ^20^

#### 2.2.4 Antinuclear antibodies assessment

Antinuclear antibodies screening was performed by the indirect immunofluorescence technique using human epithelial type 2 (HEp‐2) as a substrate (Euroimmun®). Detection of ANA at a dilution superior to or equal to 1:80 was considered a positive result.

### 2.3 Statistical analysis

Frequencies were compared using the chi‐square test or Fisher's exact test.

A correlation study between aβ2GPI‐IgA and CCP‐Ab was done by calculating the Spearman's correlation coefficient. A *p*‐value of less than 0.05 was considered statistically significant.

## 3 RESULTS

The characteristics of study sample are summarized in Table 2. We included 67 patients (43 females and 24 males) aged 29–80 years old (median age of 51 ± 14 years) and 90 HBD (56 females and 34 males, median age 37 ± 11 years). All patients were found negative for ANA. None of our patients had autoimmune diseases or hepatitis C or hepatitis B. Smoking status was not available.

**TABLE 2:** Characteristics of patients and the control group

	Patients (*n* = 67)	Control group (*n* = 90)	*p*
Sex ratio (F/M)	1.8 (43/24)	1.6 (56/34)	0.8
Mean age	51 ± 14 years	37 ± 11 years	**<10** ^**−6**^
Age range	29–80 years	20–64 years	‐

Abbreviations: F, female; M, male.

Bold values indicates the significant of *p* values.

All patients have aβ2GPI‐IgA (100%), 17.9% have aβ2GPI‐IgG and 19.4% have aβ2GPI‐IgM. For aCL, 3.3% of patients have aCL‐IgG and IgM and 7.8% have aCL‐IgA (Table 3).

**TABLE 3:** Frequency of antiphospholipid antibodies (aCL and aβ2GPI) in patients and control group

Anti‐phospholipid antibodies	Patients (*n* = 67) % (*n*)	Control group (*n* = 90) % (*n*)	*p*
aCL (IgG and/or IgA and/or IgM)	31.3 (21)	5.5 (5)	**<10** ^**−3**^
aCL‐IgG	6 (4)	2.2 (2)	**0.43**
aCL‐IgA	13.4 (9)	2.2 (2)	**0.015**
aCL‐IgM	19.4 (13)	4.4 (4)	**0.003**
aβ2GPI (IgG and/or IgA and/or IgM)	100 (67)	10 (9)	**<10** ^**−6**^
aβ2GPI‐IgG	17.9 (12)	3.3 (3)	**0.002**
aβ2GPI‐IgA	100 (67)	7.8 (7)	**<10** ^**−6**^
aβ2GPI‐IgM	19.4 (13)	3.3 (3)	**0.001**

Abbreviations: aβ2GPI, anti‐β2 glycoprotein I antibodies; aCL, anti‐cardiolipin antibodies.

Bold values indicates the significant of *p* values.

Seventeen patients and eight HBD had CCP‐Ab and/or RF (25.4% vs. 8.9%, *p* = 0.005, CI 95% [14.95; 35.79], odds ratio = 3.5). The frequency of CCP‐Ab was significantly higher in patients than in healthy subjects (14.9% vs. 3.3%, *p* = 0.009). Nine patients have only CCP‐Ab (13.4%), while only three HBD (3.3%) have this antibodies without RF (*p* = 0.02). IgA isotype of RF was significantly higher in patients than in controls (7.5% vs. 0%, *p* = 0.02). One patient (1.5%) had both CCP‐Ab and RF (Table 4). In male patients, CCP‐Ab and/or RF were more frequent than in healthy male subjects (37.5% vs. 11.8%, *p* = 0.02). CCP‐Ab were more frequent in male than in female patients (29.2% vs. 7%, *p* = 0.04; Table 5).

**TABLE 4:** Frequency of CCP‐Ab and RF in patients and in the control group

Autoantibodies	Patients (*n* = 67) % (*n*)	Control group (*n* = 90) % (*n*)	*p*	CI (95%)	Odds ratio
CCP‐Ab and/or RF	25.4 (17)	8.9 (8)	**0.005**	[14.95; 35.79]	3.5
CCP‐Ab	14.9 (10)	3.3 (3)	**0.009**	[6.39; 23.46]	1.9
Isolated CCP‐Ab	13.4 (9)	3.3 (3)	**0.02**	[5.27; 21.60]	4.5
RF (IgG and/or IgA and/or IgM)	11.9 (8)	5.5 (5)	0.15	‐	‐
RF‐IgG	6 (4)	2.2 (2)	0.23	‐	‐
RF‐IgA	7.5 (5)	0	**0.02**	[1.17; 13.76]	‐
RF‐IgM	6 (4)	5.5 (5)	>0.99	‐	‐
Isolated RF‐IgA	6 (4)	0	0.06	‐	‐
CCP‐Ab and RF	1.5 (1)	0	0.85	‐	‐

Abbreviations: CCP‐Ab, anti‐cyclic citrullinated peptides ; RF, rheumatoid factors.

Bold values indicates the significant of *p* values.

**TABLE 5:** Frequency of CCP‐Ab and RF according to sex

Autoantibodies	Females			Males		
	Patients (*n* = 43) % (*n*)	Control group (*n* = 56) % (*n*)	*p*	Patients (*n* = 24) % (*n*)	Control group (*n* = 34) % (*n*)	*p*
CCP‐Ab and/or RF	18.6 (8)	7.1 (4)	0.08	37.5 (9)	11.8 (4)	**0.02**
CCP‐Ab	7 (3)^**a**^	1.8 (1)	0.43	29.2 (7)^**a**^ ^,^ ^**b**^	5.9 (2)	**0.04**
RF (IgG and/or IgA and/or IgM)	11.6 (5)	5.4 (3)	0.44	12.5 (3)	5.9 (2)	0.67
RF‐IgG	2.3 (1)	1.8 (1)	>0.99	12.5 (3)	2.9 (1)	0.37
RF‐IgA	9.3 (4)	0	0.06	4.2 (1)^**b**^	0	0.83
RF‐IgM	4.6 (2)	5.4 (3)	>0.99	8.3 (2)	5.9 (2)	>0.99

Abbreviations: CCP‐Ab, anti‐cyclic peptides antibodies; RF, rheumatoid factors.

^**a**^ Comparison of frequencies of CCP‐Ab between male and female patients, *p* = **0.04.**

^**b**^ Comparison between frequencies of CCP‐Ab and RF‐IgA in male patients, *p* = **0.04.**

Bold values indicates the significant of *p* values.

In patients, mean titer of CCP‐Ab was 4.6 ± 6.6 RU/ml. The mean titers of RF‐IgG, IgA, and IgM were 18.8 ± 15.5 U/ml, 21.5 ± 55.6 U/ml, and 10.6 ± 32.7 UI/ml.

In patients, no correlation was found between the levels of aβ2GPI‐IgA and CCP‐Ab (*r* = 0.082, *p* = 0.51). We also did not find a correlation between the level aβ2GPI‐IgA and the level of the isotypes of RF (IgG, IgA, and IgM) in patients (*r* = 0.1, *p* = 0.37; *r* = 0.17, *p* = 0.17 and *r* = 0.07, *p* = 0.59 respectively).

## 4 DISCUSSION

In this study, we evaluated the frequency of antibodies of RA in patients admitted for suspicion of APS. The inclusion criterion of our patients was positivity of aβ2GPI‐IgA. Although the IgA isotype of aβ2GPI was not included in the classification criteria for the diagnosis of APS,^3^ some observations support the pathogenic role of these antibodies. It has been demonstrated a high binding activity of the IgA to domains IV and V of β2GPI which are associated with certain manifestations of APS.^21^, ^22^ In an experimental model of APS, aβ2GPI‐IgA has been demonstrated to induce increased thrombus formation and up‐regulate tissue factor activity.^5^, ^22^ Moreover, aβ2GPI‐IgA positivity was reported to be associated with many clinical manifestations such as myocardial infarction, atherosclerosis, acute cerebral ischemia, thrombosis, and stroke.^22^ However, aβ2GPI‐IgA can be observed in autoimmune diseases other than APS. In fact, we have previously demonstrated a significantly higher frequency of aβ2GPI‐IgA in patients with celiac disease, primary biliary cholangitis, and RA than in a healthy population.^7^, ^8^, ^9^

In this study, the frequency of antibodies of RA (CCP‐Ab or RF) was increased in patients compared with healthy subjects (25.4% vs. 8.9%, *p* = 0.005). Alessandri et al.^23^ evaluated the frequency of CCP‐Ab in patients with APS and they found that out of 79 patients, only one had CCP‐Ab. The frequency of CCP‐Ab was therefore higher in our study than in that of Alessandri et al.^23^ (14.9% and 1.3% respectively). This discrepancy could be explained by the inclusion criteria in both studies. In fact, we included patients with aβ2GPI‐IgA, while Alessandri et al.^23^ included patients with APS and for whom only the isotypes IgG and IgM have been done for both aCL and aβ2GPI.

In a previous study, we demonstrated a significantly higher frequency of aβ2GPI‐IgA in patients with RA than in healthy subjects (26.7% vs. 7.8%, *p* = 0.0007).^9^ Thanks to the present study, we discovered that the vice versa is true, which means that autoantibodies of RA (CCP‐Ab and/or RF) are significantly more frequent in patients with aβ2GPI‐IgA than controls (25.4% vs. 8.9%, *p* = 0.005). Both APS and RA are autoimmune diseases and share common risk factors. Indeed, cigarette smoking, which is known to stimulate the anti‐citrulline immunity,^24^ is a risk factor not only for RA^25^ but also for APS.^26^ Unfortunately, in Tunisia, the frequency of smoking is the highest in the world.^27^ Moreover, in our country, smoking is more frequent in men than in women.^27^ Interestingly, in the present study, the frequency of CCP‐Ab was significantly higher in males than in females (29.2% vs. 7%, *p* = 0.04). RF‐IgA was also significantly more frequent in patients than in controls (7.5% vs. 0%, *p* = 0.02). Cigarette smoking has been associated with RF‐IgA in African Americans but not in Americans with European ancestry.^25^ Remarkably, our study is about Tunisian people who are of African ancestry.

In a previous study, we evaluated the frequency of autoantibodies of RA in patients with celiac disease.^28^ Our results are similar to those of the present study. That means not only CCP‐Ab and RF were more frequent in celiac patients than in healthy subjects but also IgA was the predominant isotype of RF. So, we could imagine that our patients of the present study would develop celiac disease. Both in the present study and in our previous study on celiac disease,^28^ patients with RF‐IgA had no symptoms of RA. However, they could develop RA in the future. In fact, RF‐IgA can appear 15 years before the symptoms of RA.^29^ Indeed, RF and especially the IgA isotype was the first appearing antibody in a study, which included 321 pre‐symptomatic individuals.^29^ CCP‐Ab have also a high positive predictive value for the development of RA.^19^ The combination of CCP‐Ab and RF increases the prediction of future RA.^29^, ^30^ In the present study, 14.9% of our patients had CCP‐Ab and 11.9% had RF. However, when we combined both tests, the frequency of autoantibodies of RA increased to 25.4% in the whole group and to 37.5% in the male group.

In our laboratory, only the IgG isotype of CCP‐Ab is done, so we hypothesize that if sera of our patients could be tested in the IgA isotype of CCP‐Ab, so the global frequency of CCP‐Ab could be higher than 14.9% in the whole group and more than 29.2% in males. In fact, it has been demonstrated that 34.9% of IgG‐CCP‐Ab negative patients with RA were IgA‐CCP‐Ab positive.^31^ Moreover, it has been hypothesized that RA has mucosal origins.^32^ Among mucosal sites, which exhibit autoantibody production, the gut plays a major role in the pathogenesis of RA. Especially, gut dysbiosis has been implicated in local CCP‐Ab production.^32^, ^33^, ^34^, ^35^ In the present study, we screened for RA in patients suspected to have APS since a common link does exist between these two diseases. Interestingly, the role of gut microbiota has been described in the pathogenesis of RA^33^, ^34^ and also of APS.^36^ An increase of several species characterizes the gut dysbiosis of early RA. Among these species, Prevotella could induce an anti‐citrullinated immune response through a molecular mimicry mechanism.^35^ Moreover, in APS, cross‐reactive gut commensal antigens could induce aβ2GPI production.^37^

This study presents some limitations. First, the smoking status of patients and HBD was not specified in the medical records of the patients. Second, we do not have the lupus anticoagulant assay result for our patients. Lastly, the second sample for antiphospholipid antibodies assay was not available to confirm antiphospholipid antibody positivity 12 weeks after baseline screening.

In conclusion, in this study, we demonstrated a high frequency of autoantibodies of RA in patients with aβ2GPI‐IgA suggesting that these patients could be at higher risk of developing RA. Following the clinical features and serological markers of these patients over the years could confirm this data.

## CONFLICT OF INTEREST

None.

## Data Availability

Data are not shared.

## References

[1] McDonnell T , Wincup C , Buchholz I , et al. The role of beta‐2‐glycoprotein I in health and disease associating structure with function: more than just APS. Blood Rev. 2020;39:100610. 31471128 10.1016/j.blre.2019.100610 PMC7014586

[2] Meroni PL , Borghi MO . Antiphospholipid antibody assays in 2021: looking for a predictive value in addition to a diagnostic one. Front Immunol. 2021;12:726820. 34621272 10.3389/fimmu.2021.726820 PMC8490700

[3] Miyakis S , Lockshin MD , Atsumi T , et al. International consensus statement on an update of the classification criteria for definite antiphospholipid syndrome (APS). J Thromb Haemost. 2006;4(2):295‐306. 16420554 10.1111/j.1538-7836.2006.01753.x

[4] Rodríguez‐García V , Ioannou Y , Fernández‐Nebro A , Isenberg DA , Giles IP . Examining the prevalence of non‐criteria anti‐phospholipid antibodies in patients with anti‐phospholipid syndrome: a systematic review. Rheumatology (Oxford). 2015;54(11):2042‐2050. 26152548 10.1093/rheumatology/kev226

[5] Cabrera‐Marante O , Rodríguez de Frías E , Serrano M , et al. The weight of IgA anti‐β2glycoprotein I in the antiphospholipid syndrome pathogenesis: closing the gap of seronegative antiphospholipid syndrome. Int J Mol Sci. 2020;21(23):8972. 10.3390/ijms21238972 PMC7730063 33255963

[6] Hu C , Li S , Xie Z , et al. Evaluation of the diagnostic value of non‐criteria antibodies for antiphospholipid syndrome patients in a Chinese cohort. Front Immunol. 2021;12:741369. 34567005 10.3389/fimmu.2021.741369 PMC8461188

[7] Mankaï A , Achour A , Thabet Y , Manoubia W , Sakly W , Ghedira I . Anti‐cardiolipin and anti‐beta 2‐glycoprotein I antibodies in celiac disease. Pathol Biol (Paris). 2012;60(5):291‐295. 21839587 10.1016/j.patbio.2011.07.003

[8] Mankaï A , Manoubi W , Ghozzi M , Melayah S , Sakly W , Ghedira I . High frequency of antiphospholipid antibodies in primary biliary cirrhosis. J Clin Lab Anal. 2015;29(1):32‐36. 24687920 10.1002/jcla.21723 PMC6807226

[9] Melayah S , Changuel M , Mankaï A , Ghedira I . IgA is the predominant isotype of anti‐β2 glycoprotein I antibodies in rheumatoid arthritis. J Clin Lab Anal. 2020;34(6):e23217. 31967351 10.1002/jcla.23217 PMC7307372

[10] Jang S , Kwon EJ , Lee JJ . Rheumatoid arthritis: pathogenic roles of diverse immune cells. Int J Mol Sci. 2022;23(2):905. 35055087 10.3390/ijms23020905 PMC8780115

[11] Shapiro SC . Biomarkers in rheumatoid arthritis. Cureus. 2021;13(5):e15063. 34141507 10.7759/cureus.15063 PMC8205440

[12] Coutant F . Pathogenic effects of anti‐citrullinated protein antibodies in rheumatoid arthritis – role for glycosylation. Joint Bone Spine. 2019;86(5):562‐567. 30685537 10.1016/j.jbspin.2019.01.005

[13] Distler JH , Huber LC , Gay S , Distler O , Pisetsky DS . Microparticles as mediators of cellular cross‐talk in inflammatory disease. Autoimmunity. 2006;39(8):683‐690. 17178565 10.1080/08916930601061538

[14] Firestein GS , McInnes IB . Immunopathogenesis of rheumatoid arthritis. Immunity. 2017;46(2):183‐196. 28228278 10.1016/j.immuni.2017.02.006 PMC5385708

[15] Sorice M , Longo A , Capozzi A , et al. Anti‐beta2‐glycoprotein I antibodies induce monocyte release of tumor necrosis factor alpha and tissue factor by signal transduction pathways involving lipid rafts. Arthritis Rheum. 2007;56(8):2687‐2697. 17665396 10.1002/art.22802

[16] Iuliano A , Galeazzi M , Sebastiani GD . Antiphospholipid syndrome's genetic and epigenetic aspects. Autoimmun Rev. 2019;18(9):102352. 31323355 10.1016/j.autrev.2019.102352

[17] Viatte S , Barton A . Genetics of rheumatoid arthritis susceptibility, severity, and treatment response. Semin Immunopathol. 2017;39(4):395‐408. 28555384 10.1007/s00281-017-0630-4 PMC5486781

[18] Karpouzas GA , Ormseth SR , Hernandez E , Bui VL , Budoff MJ . Beta‐2‐glycoprotein‐I IgA antibodies predict coronary plaque progression in rheumatoid arthritis. Semin Arthritis Rheum. 2021;51(1):20‐27. 33360226 10.1016/j.semarthrit.2020.10.003

[19] Sghiri R , Bouajina E , Bargaoui D , et al. Value of anti‐mutated citrullinated vimentin antibodies in diagnosing rheumatoid arthritis. Rheumatol Int. 2008;29(1):59‐62. 18496693 10.1007/s00296-008-0614-8

[20] Sghiri R , Bouagina E , Zaglaoui H , et al. Diagnostic performances of anti‐cyclic citrullinated peptide antibodies in rheumatoid arthritis. Rheumatol Int. 2007;27(12):1125‐1130. 17447069 10.1007/s00296-007-0351-4

[21] Serrano M , Martinez‐Flores JA , Norman GL , Naranjo L , Morales JM , Serrano A . The IgA isotype of anti‐β2 glycoprotein I antibodies recognizes epitopes in domains 3, 4, and 5 that are located in a lateral zone of the molecule (L‐shaped). Front Immunol. 2019;10:1031. 31134087 10.3389/fimmu.2019.01031 PMC6515947

[22] Murthy V , Willis R , Romay‐Penabad Z , et al. Value of isolated IgA anti‐β2‐glycoprotein I positivity in the diagnosis of the antiphospholipid syndrome. Arthritis Rheum. 2013;65(12):3186‐3193. 23983008 10.1002/art.38131 PMC4048705

[23] Alessandri C , Agmon‐Levin N , Conti F , et al. Anti‐mutated citrullinated vimentin antibodies in antiphospholipid syndrome: diagnostic value and relationship with clinical features. Immunol Res. 2017;65(2):524‐531. 28215033 10.1007/s12026-017-8899-x

[24] Perricone C , Versini M , Ben‐Ami D , et al. Smoke and autoimmunity: the fire behind the disease. Autoimmun Rev. 2016;15(4):354‐374. 26772647 10.1016/j.autrev.2016.01.001

[25] Mikuls TR , Hughes LB , Westfall AO , et al. Cigarette smoking, disease severity and autoantibody expression in African Americans with recent‐onset rheumatoid arthritis. Ann Rheum Dis. 2008;67(11):1529‐1534. 18198196 10.1136/ard.2007.082669 PMC2731992

[26] Binder SR , Litwin CM . Anti‐phospholipid antibodies and smoking: an overview. Clin Rev Allergy Immunol. 2017;53(1):1‐13. 27377297 10.1007/s12016-016-8565-4

[27] Fakhfakh R , Hsairi M , Maalej M , Achour N , Nacef T . Tobacco use in Tunisia: behaviour and awareness. Bull World Health Organ. 2002;80:350‐356. 12077609 PMC2567801

[28] Ghozzi M , Melayah S , Adaily N , Ghedira I . Frequency of serological markers of rheumatoid arthritis in adult patients with active celiac disease. J Clin Lab Anal. 2022;36(3):e24249. 35060192 10.1002/jcla.24249 PMC8906036

[29] Brink M , Hansson M , Mathsson‐Alm L , et al. Rheumatoid factor isotypes in relation to antibodies against citrullinated peptides and carbamylated proteins before the onset of rheumatoid arthritis. Arthritis Res Ther. 2016;18:43. 26860413 10.1186/s13075-016-0940-2 PMC4748586

[30] Greenblatt HK , Kim HA , Bettner LF , Deane KD . Preclinical rheumatoid arthritis and rheumatoid arthritis prevention. Curr Opin Rheumatol. 2020;32(3):289‐296. 32205569 10.1097/BOR.0000000000000708 PMC7340337

[31] Sokolova MV , Hagen M , Bang H , Schett G , Rech J , Steffen U . RETRO study group. IgA anti‐citrullinated protein antibodies (IgA ACPA) are associated with flares during DMARD tapering in rheumatoid arthritis. Rheumatology (Oxford). 2021;61:2124‐2131. 10.1093/rheumatology/keab585 34508547

[32] Holers VM , Demoruelle MK , Kuhn KA , et al. Rheumatoid arthritis and the mucosal origins hypothesis: protection turns to destruction. Nat Rev Rheumatol. 2018;14(9):542‐557. 30111803 10.1038/s41584-018-0070-0 PMC6704378

[33] Horta‐Baas G , Romero‐Figueroa MDS , Montiel‐Jarquín AJ , Pizano‐Zárate ML , García‐Mena J , Ramírez‐Durán N . Intestinal dysbiosis and rheumatoid arthritis: a link between gut microbiota and the pathogenesis of rheumatoid arthritis. J Immunol Res. 2017;2017:4835189. 28948174 10.1155/2017/4835189 PMC5602494

[34] Zaiss MM , Joyce Wu HJ , Mauro D , Schett G , Ciccia F . The gut‐joint axis in rheumatoid arthritis. Nat Rev Rheumatol. 2021;17(4):224‐237. 33674813 10.1038/s41584-021-00585-3

[35] Lucchino B , Spinelli FR , Iannuccelli C , Guzzo MP , Conti F , Di Franco M . Mucosa‐environment interactions in the pathogenesis of rheumatoid arthritis. Cell. 2019;8(7):700. 10.3390/cells8070700 PMC6678242 31295951

[36] Ruff WE , Vieira SM , Kriegel MA . The role of the gut microbiota in the pathogenesis of antiphospholipid syndrome. Curr Rheumatol Rep. 2015;17(1):472. 25475595 10.1007/s11926-014-0472-1 PMC4394866

[37] Ruff WE , Kriegel MA . Autoimmune host‐microbiota interactions at barrier sites and beyond. Trends Mol Med. 2015;21(4):233‐244. 25771098 10.1016/j.molmed.2015.02.006 PMC5918312

